# Analysis of Major Depression Risk Genes Reveals Evolutionary Conservation, Shared Phenotypes, and Extensive Genetic Interactions

**DOI:** 10.3389/fpsyt.2021.698029

**Published:** 2021-07-15

**Authors:** Saveen Sall, Willie Thompson, Aurianna Santos, Donard S. Dwyer

**Affiliations:** ^1^Department of Psychiatry and Behavioral Medicine, Louisiana State University Health Shreveport, Shreveport, LA, United States; ^2^Department of Pharmacology, Toxicology and Neuroscience, Louisiana State University Health Shreveport, Shreveport, LA, United States

**Keywords:** *C. elegans*, evolutionary conservation, gene-gene interactions, genetic risk factor, major depressive disorder, zebrafish

## Abstract

Major depressive disorder (MDD) affects around 15% of the population at some stage in their lifetime. It can be gravely disabling and it is associated with increased risk of suicide. Genetics play an important role; however, there are additional environmental contributions to the pathogenesis. A number of possible risk genes that increase liability for developing symptoms of MDD have been identified in genome-wide association studies (GWAS). The goal of this study was to characterize the MDD risk genes with respect to the degree of evolutionary conservation in simpler model organisms such as *Caenorhabditis elegans* and zebrafish, the phenotypes associated with variation in these genes and the extent of network connectivity. The MDD risk genes showed higher conservation in *C. elegans* and zebrafish than genome-to-genome comparisons. In addition, there were recurring themes among the phenotypes associated with variation of these risk genes in *C. elegans*. The phenotype analysis revealed enrichment for essential genes with pleiotropic effects. Moreover, the MDD risk genes participated in more interactions with each other than did randomly-selected genes from similar-sized gene sets. Syntenic blocks of risk genes with common functional activities were also identified. By characterizing evolutionarily-conserved counterparts to the MDD risk genes, we have gained new insights into pathogenetic processes relevant to the emergence of depressive symptoms in man.

## Introduction

Major depressive disorder (MDD) is a distressing psychiatric condition characterized by chronic sadness, hopelessness, anhedonia, labile affect, sleep disturbances, and psychotic features in severe forms (1). Consequently, it adversely affects quality of life in various dimensions (2, 3), but also elevates the risk of suicide (4). In fact, MDD contributes to roughly half of all suicide attempts and increases the risk of suicide to nearly 20% when left untreated (5). This disorder has a lifetime prevalence of about 15–18% and is nearly twice as common in females as males (6, 7). Genetic factors clearly contribute to individual liability for MDD (8–10); however, heritability is somewhat less (30–40%) compared to other psychiatric disorders such as schizophrenia (65–80%) and bipolar disorder (60–80%) (11–14). Based on these heritability data, genome-wide association studies (GWAS) and candidate gene analyses have sought to identify genes (risk variants) that increase the probability of developing MDD. Collectively, these studies have now identified several hundred variants with solid evidence for their consideration as viable risk genes (8, 10, 15–26).

Findings from GWAS analysis may help to fill in gaps in our knowledge about the pathogenesis of MDD. Currently, the major hypothesized mechanisms focus on altered activity of neurotransmitters (e.g., monoamines and acetylcholine), growth factor signaling (especially brain-derived neurotrophic factor, BDNF), immune system components (cytokines), defective regulation of the hypothalamus-pituitary-adrenal (HPA) axis and environmental factors including childhood trauma and stress (1, 27–29). Presumably, the genetic risk variants affect the function of these pathways directly and/or else the resiliency phenotype needed to cope with adverse psychosocial impacts.

If the GWAS data are to inform us about causative factors in MDD, we must have confidence in both their identification and meaning. Larger GWAS analyses involving tens of thousands of cases and controls, along with technical improvements and statistical refinement, have provided increasingly trustworthy findings concerning risk variants. However, the validity of putative risk genes would be further strengthened by showing that they: (1) connect in a meaningful way to the pathogenesis of MDD, (2) mediate common or shared phenotypes, and (3) interact with each other at some level (e.g., genetic or protein-protein interactions). Furthermore, we may gain insights into the function of the risk genes by exploring their activity and associated phenotypes in model organisms such as *Caenorhabditis elegans* and zebrafish.

To search for common molecular mechanisms and relationships between the MDD risk genes identified so far, we assembled a list of 336 genes derived from numerous GWAS analyses (8, 10, 15–26). The genes were characterized in terms of degree of evolutionary conservation, the phenotypes associated with their genetic counterparts in *C. elegans*, and gene-gene interactions. Based on previous findings in schizophrenia (30) and bipolar disorder (31), we hypothesized that the MDD risk genes would show an increase in evolutionary conservation and genetic interactions with one another in comparison to randomly-selected genes. As reported here, the MDD risk genes are highly conserved during evolution, involved in essential and pleiotropic processes and are extensively linked in gene interaction networks. Moreover, we identified blocks of risk genes that appear to operate together toward common functions. Analysis of phenotypes and functional activities associated with the *C. elegans* counterparts of the MDD risk genes revealed molecular mechanisms possibly involved in the pathogenesis of depression. The data supported a role for BDNF and insulin/IGF-1 signaling in MDD and highlighted the potential contributions of additional genes, including the LHX family of transcription factors and kinases such as MARK2/3 and BRSK2.

## Materials and Methods

### MDD Risk Gene Compilation

In order to characterize genes implicated as risk factors for MDD, we first compiled a list of genes derived from primary studies (GWAS) and meta-analyses by the following investigators: (8, 10, 15–26). After removing any duplicates, we created a final list of 336 genes summarized in Supplementary Table 1. The table includes brief gene descriptors, chromosomal locations, associated variants where available, genetic counterparts in *C. elegans* and zebrafish, and phenotypes derived from the *C. elegans* database, WormBase (32). We focused on these two species to be comparable to prior research on other psychiatric disorders (30, 31), to span a broad evolutionary time-scale and to take advantage of the rich phenotype data available for *C. elegans* genes. Finally, we noted all of the MDD risk genes that did not have a counterpart in *C. elegans* (33) and zebrafish (12) by inserting “None” for that species in Supplementary Table 1.

### Identification of Orthologs and Functional Counterparts of the MDD Risk Genes in *C. elegans* and Zebrafish

As a starting point, we entered the human gene designation in the query box of the Ensembl website [maintained by the European Bioinformatics Institute, EMBL-EBI; (34)] and searched the list of orthologs for established counterparts in *C. elegans* and zebrafish. These orthologs were then recorded in Supplementary Table 1. Some orthologs of human genes are not listed on the Ensembl website, even though a search starting at WormBase can identify human orthologs of the *C. elegans* genes missed by Ensembl. The same is true for some zebrafish genes. Roughly 30% of the MDD risk genes failed to list a *C. elegans* ortholog. In these instances, we chose the transcript with the longest amino acid sequence to search the WormBase site (*C. elegans*) or the Ensembl site (zebrafish) with BLAST. Counterpart genes were added to the list when the proteins they encoded showed >20–25% identity with the query sequence over extended segments in proteins of similar lengths and based on convergence of searches using the sequences of other species (e.g., anole lizard, golden hamster, and spotted gar) on the same gene product. In a few cases, the paralog of the risk gene was used instead for the BLAST search according to this same scheme. These cases have been noted in the table. In 30 of 106 cases, the BLAST search found a *C. elegans* gene already recognized as the human counterpart at WormBase, presumably due to differences in curation of the databases. In 60 cases, no counterpart was found including a distant homolog. Only 16 (6%) of the human gene counterparts in *C. elegans* listed in Supplementary Table 1 were more distant homologs of the human risk genes. For zebrafish, 37 genes were subjected to this type of analysis and only 4 are considered distant homologs (20–25% identity). Therefore, the vast majority of the risk gene counterparts in *C. elegans* and zebrafish are widely recognized as the corresponding gene products.

Because WormBase is a comprehensive source of information concerning the *C. elegans* genes, we retrieved the phenotypes associated with the counterparts of the MDD risk genes and included these phenotypes in the master list in Supplementary Table 1. The phenotypes were the result of natural and induced genetic variation or RNA interference (RNAi) with normal gene expression.

### Characterization of Syntenic Blocks of Risk Genes

Previously, we (30, 31) identified blocks of risk genes for schizophrenia and bipolar disorder that were co-localized near a disease risk variant and that appeared to serve a common function or purpose. These same genes were typically located near to each other in zebrafish and *C. elegans*, but were in closest proximity in humans. These types of gene arrangements were termed syntenic blocks of genes to reflect their conserved chromosomal localization and common functional activity. Using the criteria described elsewhere (31), we searched for similar syntenic blocks of genes associated with the MDD risk variants. Twenty-three blocks met the criteria (see Table 1). We then characterized these gene blocks with respect to shared phenotypes derived from the *C. elegans* counterparts and in terms of their functional activity. The functions of the gene blocks were established from two major sources: the gene ontology (GO) information from the Ensembl website and from PubMed (National Library of Medicine) searches using the human gene designation for the query.

**Table 1:** Characterization of syntenic blocks of genes.

	**Genes**	**Location human**	**Location zebrafish**	**Shared phenotypes**	**Functional activity**
1	SYPL2-CYB561D1-GNAI3-GNAT2-AMPD2	1p13.3	8, 11	Locomotion 3/5 Lethal 2/5 Roaming increased 2/5	GTP production/G-protein signaling Brain development **BDNF connections**
2	MYOC-VAMP4-EEF1AKNMT-DNM3	1q24.3	20	Lethal 4/4 Life span 2/4 Aldicarb resistant 2/4	Synaptic vesicle turnover Growth/proliferation
3	**CRB1-DENND1B-**C1orf53**- LHX9**	1q31.3	22	Lethal 2/4 Sterile 2/4 Development 2/4	Cell polarity Neuronal migration/ differentiation
4	ANKRD44-SF3B1-COQ10B-HSPD1-HSPE1-MOB4-RFTN2-MARS2-BOLL-PLCL1	2q33.1	9, 19	Lethal 5/9 Sterile 5/9 Transgene variant 4/9 Life span 4/9	RNA binding/splicing Mitochondrial function **Insulin/Akt signaling** Synaptic function
5	**SHOX2**-RSRC1-MLF1-LXN-GFM1-RARRES1-MFSD1	3q25.32	15	None 5/7 Transgene variant 2/7	DNA/RNA binding Regulate transcription/translation Developmental delay
6	TMEM33-DCAF4L1-SLC30A9-BEND4	4p13	14, 20	None 3/4	DNA/chromatin binding
7	LHFPL2-AP3B1-SCAMP1	5q14.1	21	None 2/3	Development reproductive tract Fertilization
8	SIM1-GRIK2-ASCC3 … HACE1-LIN28B-BVES	6q16.3	16, 20	Dauer life span 2/6	DNA/RNA binding Cell migration/differentiation Neuronal development
9	SP4 … NPY-MPP6-GSDME-OSBPL3	7p15.3	19	Lethal 3/4 Embryonic dev. 3/4 Locomotion 2/4	Nervous system development **BDNF connections** **Ketamine response**
10	**PAX5**-ZCCHC7-GRHPR-ZBTB5-POLR1E	9p13.2	1, 17	Lethal 5/5 Sterile 4/5 Slow growth 3/5	DNA/RNA binding Regulation of transcription Immune/Nervous system development
11	ASTN2-TLR4 … **(CRB2)-DENND1A-LHX2**	9q33.1-3	5, 13, 21	Development 3/5 Lethal 2/5	Synaptic function Brain development/autism
12	ARL3-SFXN2-WBP1L-CYP17A1-BORCS7-AS3MT-CNNM2-NT5C2	10q24.32	1, 13	Transgene variant 3/7	Metal binding/homeostasis Brain development Schizophrenia
13	DCDC1-DNAJC24-IMMP1L-ELP4-**PAX6**-RCN1-CCDC73-EIF3M-PRRG4-QSER1-(**HIPK3**)	11p13	7, 18, 25	Lethal 4/8 Sterile 2/8	Transcription factor/RNA regulation Ca^++^/ion binding Apoptosis
14	**DRD4 … (BRSK2) … (INS/IGF2)-TH … KCNQ1 …** NUP98**-**PGAP2**-STIM1**	11p15.4-5	7, 21, 25	Body posture 3/6 Lethal 2/6 Life span 2/6	Neurotransmission **Insulin/Akt signaling** Brain development and disorders **BDNF connections**
15	DAGLA-MYRF-TMEM258-FADS1-FADS2-FEN1-FADS3-RAB3IL1	11q12.2-3	7, 24, 25	Slow growth 5/8 Locomotion 5/8 Life span 4/8 Sluggish 4/8	Lipid metabolism Myelination
16	**RTN3**-C11orf95-SPINDOC-**MARK2**-RCOR2	11q13.1	7	None 5/5	Regulation of transcription Wnt signaling Neuronal development/migration
17	TTC12-ANKK1-**DRD2**-TMPRSS5	11q23.2	15	Lethal 3/4 Sterile 3/4 Dauer defect 2/4 Locomotion 2/4	**Insulin/BDNF connections** Substance use disorders
18	POP5-CABP1-MLEC-UNC119B-ACADS-SPPL3-OASL-CAMKK2-ANAPC5-RNF34-KDM2B	12q24.31	5, 8, 10	Lethal 7/10 Embryonic dev. 5/10 Pharyngeal pumping 2/10	Immune function Schizophrenia **BDNF connections**
19	CCDC175-**RTN1**-LRRC9-PCNXL4-DHRS7-PPM1A	14q23.1	13, 17	None 6/6	TGF-retinoid crosstalk Neuronal differentiation BRSK2 regulation
20	LTBP2-ISCA2-AREL1-FCF1-YLPM1-PROX2-DLST-RPS6KL1	14q24.3	17, 20	Slow growth 4/6 Lethal 3/6 Locomotion 3/6 Fat reduced 2/6	DNA/RNA binding & regulation Energetics/catabolism Neuronal development
21	**MARK3**-CKB-TRMT61A-BAG5-COA8-KLC1-XRCC3	14q32.32-33	13, 20	Lethal 4/6 Sterile 3/6 Reduced brood 3/6	Apoptosis Microtubule function
22	XPNPEP3-EP300-L3MBTL2-CHADL-RANGAP1-POLR3H … TCF20-NFAM1-RRP7A-SERHL2	22q13.2	1, 3, 12	Slow growth 5/8 Germ cell dev. 4/8 Rachis morphology 3/8 Lethal 3/8	DNA/RNA binding β-catenin signaling Neurogenesis
23	MOV10L1-PANX2-TRABD-SELENOO-TUBGCP6-HDAC10-MAPK12-MAPK11-PLXNB2-DENND6B	22q13.33	4, 6, 18, 25	Protein expression variant 4/8 Life span 2/8 Oxidative stress 2/8	Microtubule function Cell migration/development Cell cycle

*Genes from Supplementary Table 1 are grouped here according to blocks based on their co-localization on both human and zebrafish chromosomes. Parentheses mark genes that were not included with the risk genes originally, but are within or immediately adjacent to the blocks of MDD risk genes. Ordinarily, the genes in a block flank or overlap each other physically, whereas greater separation has been indicated with ellipsis points. Noteworthy genes, including paralogs, and recurring functional activities (see text) are emphasized in bold font. “Shared phenotypes” refers to the number of genes in a block that are associated with a particular phenotype. For example, in block 2, all four genes cause lethality, 2 of 4 affect life span and 2 of 4 genes confer resistance to aldicarb, a cholinesterase inhibitor. In block 6, 3 of the 4 genes have no reported phenotype in C. elegans*.

### Quantification of Gene-Gene Interactions Among the MDD Risk Genes

Genetic interactions among risk genes provide valuable information about the interconnectivity of gene networks. To characterize gene-gene interactions in the MDD risk gene set, we employed the network prediction program GeneMANIA (35). We performed the analysis with the Networks setting restricted to “Genetic Interactions” and with “Max resultant genes” and “Max resultant attributes” set to 0. The Genetic Interaction dataset is based on the work of Lin et al. (36) that cataloged human gene interactions from the genotypes of radiation hybrid cell lines. The GeneMANIA networks were captured as figures and the number of links (connections) per gene was automatically quantified. For comparison, we generated four comparable-sized lists of randomly-selected human genes with Random Gene Set Generator from Molbiotools. The genes that were selected randomly from the genome were then analyzed in the same way with GeneMANIA to determine background (random) levels of gene-gene interactions. Because random genes do form networks based on gene-gene, protein-protein and other types of interactions, it is important to include these data as a reference point for the risk gene analysis.

### Statistical Analyses

#### Analysis of Evolutionary Conservation and Phenotype Occurrence

To determine whether the MDD risk gene counterparts showed greater conservation during evolution than the corresponding whole genomes of *C. elegans* and zebrafish, we performed chi-square analysis. Because the data were non-parametric (presence or absence of a counterpart gene when comparing human vs. model organism genomes), this was an appropriate statistical test to use. Details have been included in the figure legends. Kim et al. (37) and Howe et al. (38) previously published comparative genome analysis between humans and *C. elegans* and zebrafish, respectively. These data were used to derive the observed vs. expected frequency tables for analysis. Similarly, chi-square tests were performed to determine if differences in the frequency of phenotypes associated with genomic genes vs. MDD risk gene counterparts in *C. elegans* were significant.

#### Analysis of Gene-Gene Interactions

To evaluate the gene interaction data, we first generated four sets of randomly-selected genes as described above. These four sets were analyzed separately for Genetic Interactions with GeneMANIA and the total number of links per gene was quantified. The data from these four sets of genes was averaged and the standard deviation was determined to derive confidence intervals for comparison to the number of links per gene obtained with the MDD risk-gene set. A three standard deviation distance from the mean of the random gene-set data was used as the cutoff for determining significant differences (*p* <0.01).

## Results

### Evolutionary Conservation of MDD Risk Genes

**Figure 1:**
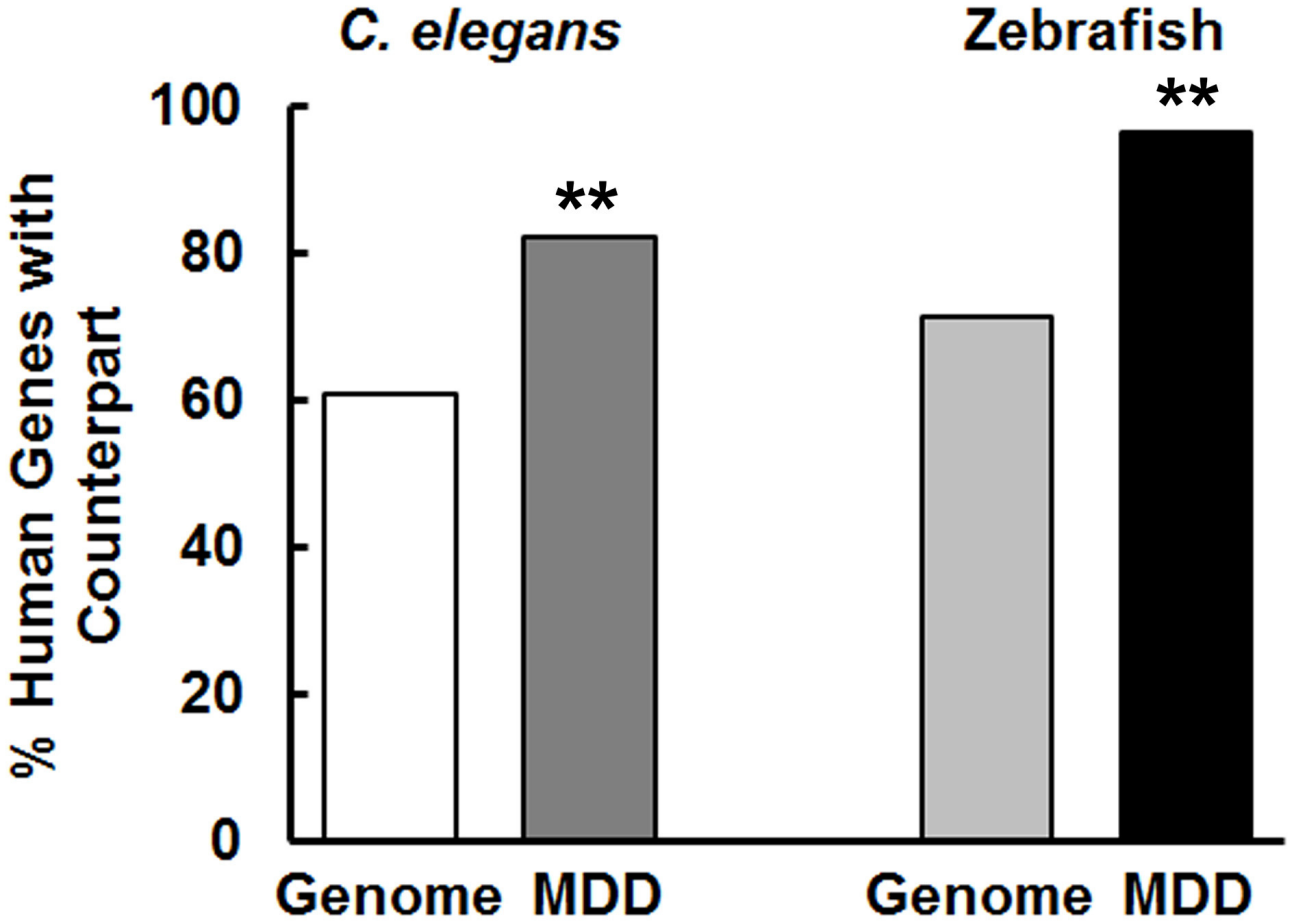
MDD risk genes are evolutionarily conserved across species. In contrast to genome-to-genome comparisons, there was significantly greater sharing of MDD risk genes between humans and *C. elegans* and zebrafish (**p < 0.01). For conservation comparisons, 12345/20310 (60.8%) of *C. elegans* genes were orthologs of human genomic genes, whereas 276/336 (82.1%) of MDD genes had counterparts in *C. elegans*. In zebrafish, the corresponding numbers were 14623/20479 (71.4%) and 324/336 (96.4%) for genomic genes and MDD risk genes, respectively. These values were used in the chi-square analysis to determine statistical significance. Because we used the entire population of data, there are no error bars.

At an intuitive level, one might imagine that genes causing MDD—a disorder of higher brain function affecting extensive neural networks including the limbic system—would tend to be unique to humans or more prevalent in higher species and absent in simple organisms that lack equivalent brain regions and behavioral complexity. However, based on previous work by our group (30, 31), we hypothesized that MDD risk genes would be evolutionarily conserved. As summarized in Figure 1, the MDD risk-gene counterparts are highly conserved (*p* <0.01) in *C. elegans* in comparison to genomic analyses of human genes found in this species. Thus, Kim et al. (37) reported that 60.8% of human genes have an ortholog in *C. elegans* compared to 82.1% of MDD genes as shown here. Similar conservation of MDD risk genes was observed in zebrafish. Across the entire genome, 71.4% of human genes had an ortholog (38), whereas 96.4% of MDD genes had a counterpart in this species (Figure 1). These data are consistent with findings of strong conservation of risk genes for schizophrenia and bipolar disorder in these two species. In fact, the degree of conservation of MDD risk genes is midway between that observed in the other two psychiatric disorders.

### Phenotypes Associated With MDD Risk-Gene Counterparts in *C. elegans*

**Figure 2:**
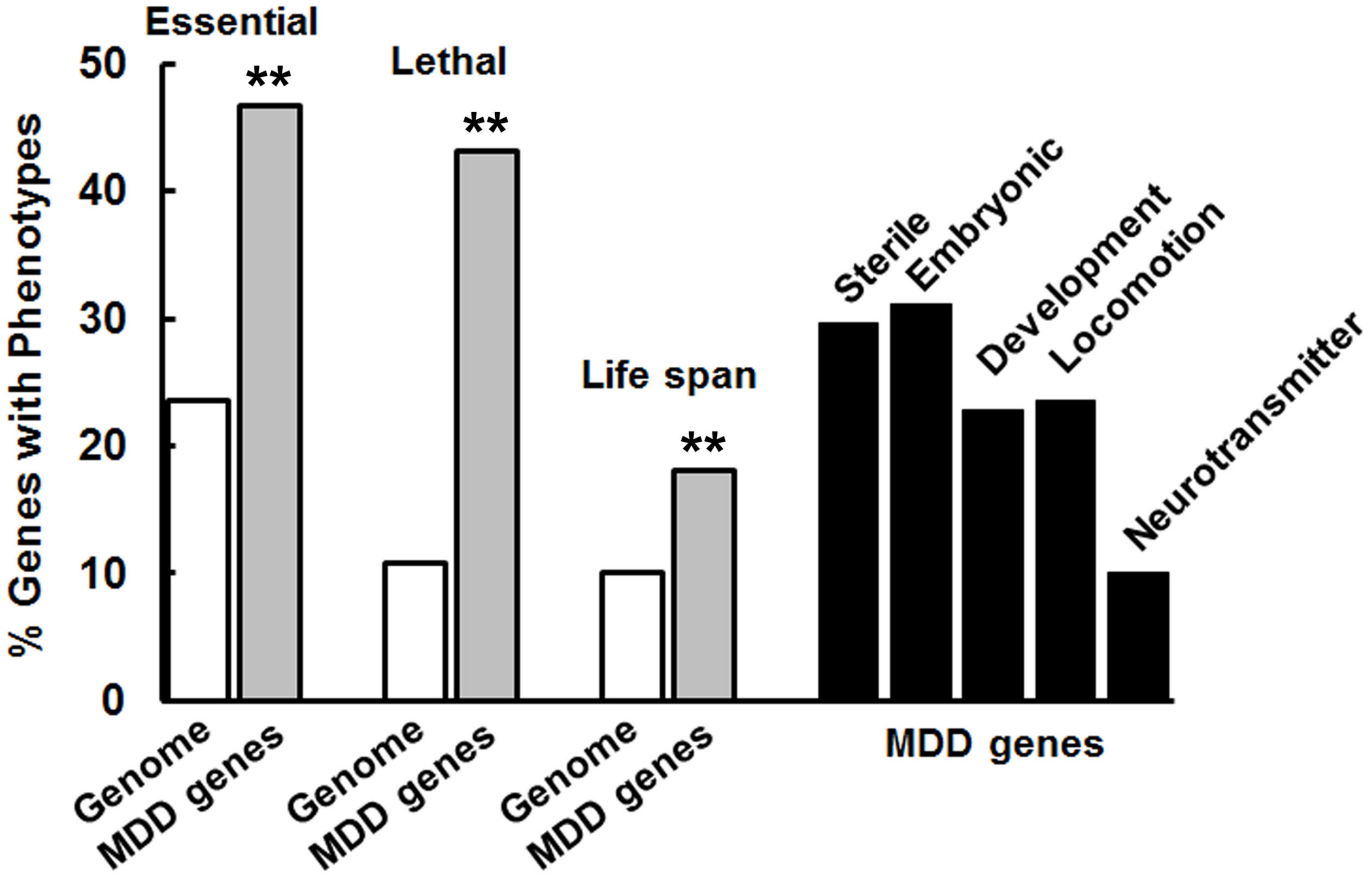
MDD risk genes are enriched for genes that are essential for life and affect life span. Essential, Lethal, and Life span phenotypes were significantly more frequent (**p < 0.01) among the risk genes than genes in general (Genome analysis) as determined by chi-square analysis similar to Figure 1. Different studies showed that 4645/19727 *C. elegans* genes are considered essential, 264/2445 of those evaluated caused lethality and 1876/18496 affected life span. For the MDD genes, 129/276 (276 is the number of genes with counterparts in *C. elegans*) were essential, 119/276 caused lethality and 50/276 affected life span. These values were used in the chi-square tests. A wide variety of phenotypes (labeled) is associated with variation in the counterpart genes in *C. elegans* and many of these genes produced more than a single phenotype reflecting their pleiotropic effects.

More than 18,000 genes have been extensively characterized in *C. elegans* for phenotypes associated with genetic variation or that were observed in RNAi studies. This information can provide initial insights into the biological activity of genes under investigation as possible causative factors in disease. Consequently, we characterized the phenotypes associated with the set of MDD risk genes. The data are summarized in Figure 2, along with information about the frequency of essential and lethal genes in the human genome for comparison. Overall, 82% (276/336) of the MDD risk-gene counterparts produced observable phenotypes in *C. elegans*. In comparison to whole or partial genome analyses, the MDD risk genes are significantly enriched for genes that are considered essential for life (producing lethality and/or sterility when altered). Of course, some of the genes that are essential or lethal upon mutation in *C. elegans* may not produce severe phenotypes when mutated in humans because of redundancy, diversification of function or other reasons. Nevertheless, the enrichment of MDD risk genes for these critical functions distinguishes this population of genes. Furthermore, genes that affect life span are significantly over-represented in the MDD risk-gene set compared to genes in general. Prominent phenotypes could also be categorized as to whether they affect early (Sterile, Embryonic), middle (Development, non-embryonic), or later stages (Locomotion, Neurotransmitter) of organism development (Figure 2). In general, the MDD risk-gene counterparts appear to be very pleiotropic and participate in a broad array of critical functions that manifest over the lifetime of the organism.

### Characterization of Gene-Gene Interactions Among the MDD Risk Genes

**Figure 3:**
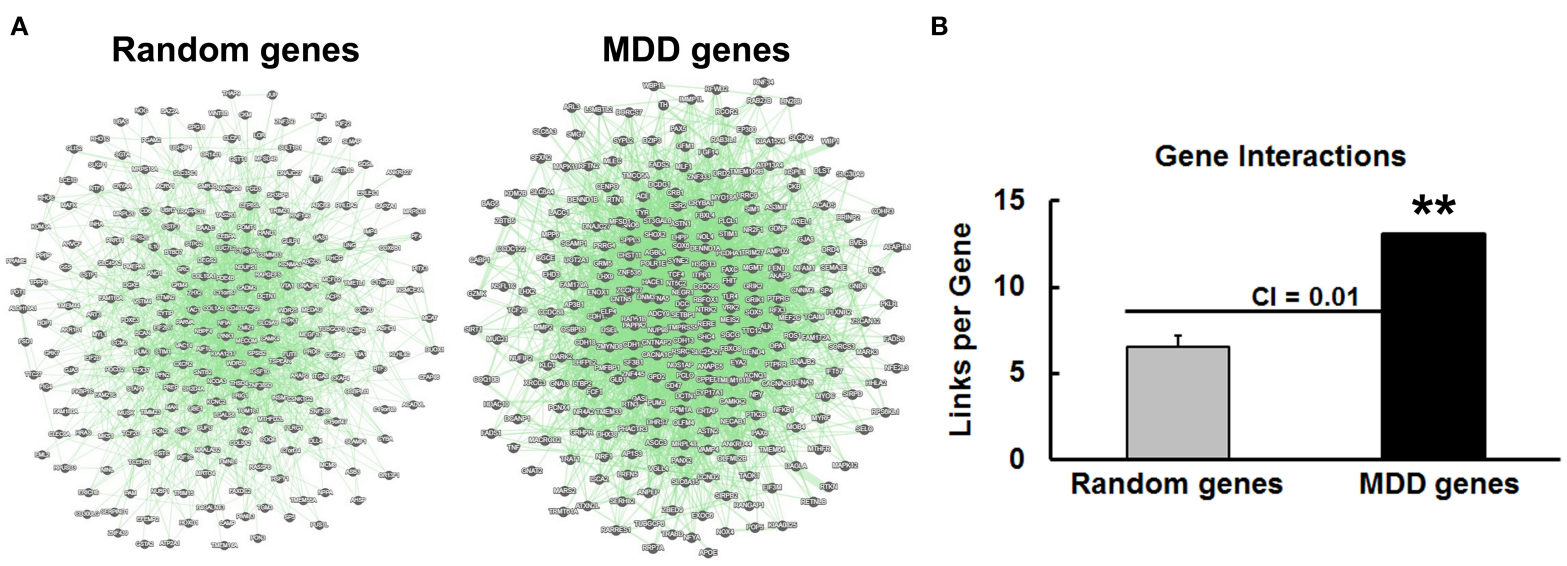
Genetic interactions among MDD risk genes. **(A)** The interaction network involving a randomly-selected set of genes is shown at the left side in comparison to the network obtained with the MDD risk genes. Green lines represent links or connections between the genes represented as gray circles. **(B)** Gene-gene interactions (Links per Gene) were automatically calculated with GeneMANIA for four lists of randomly-selected genes vs. the MDD gene set. The mean and standard deviation were depicted for the Random genes along with the confidence interval (CI) at 0.01. There were significantly more connections (**p < 0.01) among the MDD risk genes than the random genes.

Another potential contributor to the overall burden of genetic liability of risk genes would be their degree of connectedness in gene networks. Genetic interactions can amplify the small effects of individual risk variants and facilitate co-regulation of expression and activity of groups of connected genes. We analyzed genetic interactions among the MDD risk genes with GeneMANIA in comparison to background levels of interaction among four sets of randomly-selected genes. Gene interaction networks created with GeneMANIA are depicted in Figure 3A and show many connections for both the MDD risk genes and one example of the randomly-selected genes. When the links or connections per gene are quantified, the data reveal that the MDD risk genes make significantly more connections with other genes in this set than the number of connections per gene among the random genes in a set (Figure 3B). On average, the random genes make 6.5 connections with other members of the set, whereas this number is doubled for the MDD risk genes. Previous studies by our group (30, 31) revealed that the number of connections per gene for risk genes implicated in schizophrenia and bipolar disorder were 8 and 22, respectively.

**Figure 4:**
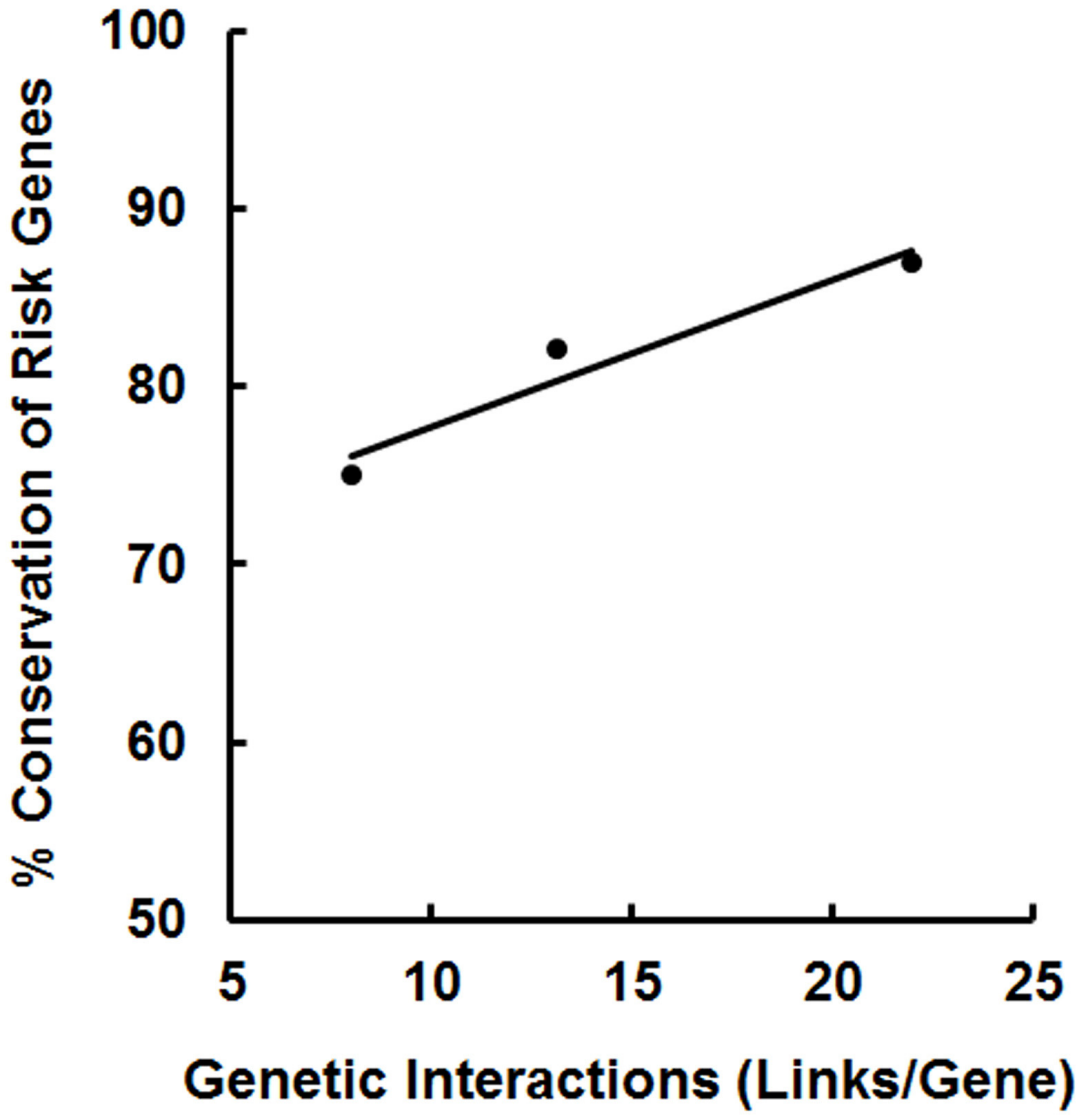
Correlation between the number of gene-gene interactions and the degree of conservation of the risk genes for schizophrenia [left data point; (30)], MDD (middle point; this study) and bipolar disorder [right data point; (31)]. Each data point is derived from a different study of the extent of conservation of disease risk genes in *C. elegans*: 257/344 (75%; schizophrenia), 276/336 (82%; MDD), and 199/230 (87%; bipolar disorder) plotted against the number of gene-gene interactions found for the different sets of risk genes. A correlation coefficient of 0.97 was obtained for these data.

Taking the data from these three studies together, an interesting pattern emerges: the degree of conservation of the risk genes during evolution is highly correlated (*r* = 0.97) with the number of links per gene from the genetic interaction analysis (Figure 4). It appears that gene-gene interactions increase as a function of the evolutionary age of a gene; thus, older genes acquire more connections. The genetic interactions may have important functional consequences because they may extend to risk genes found in syntenic blocks (next section) that can be jointly regulated.

### Identification of Syntenic Blocks of MDD Risk Genes

When we compiled the risk variants from 14 different groups, we noticed that some of the variants clustered at similar chromosomal locations. In other instances, the gene affected by a risk variant could not be resolved at the single-gene level; many in the vicinity might be disorder-associated genes. These observations resembled the situation previously noted for risk genes in other psychiatric disorders (30, 31), which led to the discovery of syntenic blocks of genes ostensibly brought together during evolution to serve a common function (30, 39, 40). Therefore, we examined the panel of 336 risk genes for evidence of syntenic arrangements of these genes across species. Out of 36 candidates, 23 merited consideration as syntenic blocks of genes and these have been summarized in Table 1.

If indeed these groups of genes serve a similar purpose, we would expect to see shared phenotypes associated with them and common functional activities including gene ontology (GO) terms. Several points are noteworthy. (1) Genes that are paralogs or family members occur in multiple blocks—most strikingly compare the composition of block 3 with block 11. Both contain CRB genes, DENND1 paralogs and members of the LHX family of transcription factors. Likewise, the kinase paralogs MARK2 and MARK3 are found in blocks 16 and 21. (2) In many cases, the genes within a syntenic block cause similar phenotypes when they are altered, for example all of the genes in blocks 2 and 10 cause lethality, 5/8 in blocks 15 and 22 cause slow growth and 3/4 in block 9 affect embryonic development. (3) Recurring themes were evident when examining common GO terms or functional activities turned up in database searches as indicated in the last column. Moreover, this analysis revealed mechanistic connections to established pathways implicated in the pathogenesis of MDD such as BDNF (many blocks), insulin signaling and even the response to ketamine. Block 14 is very intriguing because the genes listed here span a greater distance and include a dopamine receptor (DRD4), the enzyme responsible for dopamine synthesis (TH), together with STIM1 and KCNQ1 involved in insulin secretion and activity.

## Discussion

By characterizing MDD risk genes in model organisms, we were able to discern important features and functional activities of these genes. In agreement with our initial hypotheses, the risk genes were extensively conserved across species, were enriched for essential, pleiotropic genes and were highly interconnected with one another. Furthermore, the cross-species comparisons led to identification of co-localized blocks of genes with syntenic arrangements—especially when comparing human and zebrafish genomes—with shared biological functions. The syntenic blocks of genes identified here are similar to functional gene clusters described previously by several groups (40–43). Lee and Sonnhammer (42) found that genes in various metabolic pathways are located closer to each other in the genome than expected by chance and form cooperative blocks. Importantly, Friederichs et al. (43) reported that three disease (cardiomyopathy) risk genes cosegregated during evolution as discovered, in part, through comparisons with the gene arrangement in zebrafish, which is very similar to our observations. The cosegregating disease genes were located in a linkage disequilibrium block; such blocks span around 50-60 kb on average in humans (44), but are generally smaller in model organisms including *Caenorhabditis* and *Drosophila* (45, 46). Despite a much greater average distance between random pairs of genes in humans vs. model organisms (42), the genes in the syntenic blocks are in closer proximity in humans reflecting compression of these regions over time. Syntenic gene clusters may have been formed in a process of adaptive gene relocation (47). Insights from this study were only possible because we explored the chromosomal locations, functional activities and phenotypes associated with the MDD risk genes in model organisms, including *C. elegans* and zebrafish.

The findings reported here for the MDD risk genes complement previous work aimed at characterizing risk genes for schizophrenia (30) and bipolar disorder (31). In all of these psychiatric disorders, the risk genes are conserved during evolution to a significantly greater degree than genomic genes and are highly enriched for genes that are essential for life and normal development. Because of these properties, the risk variants are likely to reside in less dynamic regions of the genome known as recombination coldspots (48, 49) and may have a low chance of negative selection (50, 51). Consequently, these variants represent an ongoing liability for MDD at some recurring frequency in the human population.

Identification of MDD risk genes has relied heavily on data from GWAS analysis (8, 18, 19, 23), which has its limitations. The risk variants provide a chromosomal position of interest; however, there may be many genes in the vicinity of the marker or alternatively no genes nearby. Most often, it is the former case that then raises the question of which gene(s) are the actual causative factors for the disorder. The present work potentially adds credence to the validity of many of the MDD risk genes listed in Supplementary Table 1. First, we found that numerous putative risk genes operate in pathways linked to the pathogenesis of MDD such as BDNF, synaptic function and brain development. We would not expect to distinguish common pathways if many of the putative risk genes were actually false positives from GWAS. Second, in the syntenic blocks of genes identified here (Table 1), many gene variants produced identical phenotypes in *C. elegans*. For example, in blocks 2 and 10, genetic variation in all of the co-localized genes produced lethality in *C. elegans*. In other blocks, the majority of the risk genes caused the same phenotype when altered: e.g., 3/5 locomotion (block 1), 5/9 sterile (block 4), 3/5 development (block 11), and 5/8 slow growth (block 15). This is highly unlikely to be the result of coincidence. Lastly, the MDD risk genes were highly interactive, much more so than randomly-selected genes. This network connectivity is characteristic of risk genes for other psychiatric disorders (30, 31), which increases our confidence that most of the risk genes compiled here are indeed relevant to MDD.

Gene interaction analysis underscored two additional important points. First, we report here for the first time, the relationship between degree of network interaction among risk genes and the extent of their evolutionary conservation. Thus, risk genes that are highly conserved also tend to interact with a larger number of fellow risk genes. This makes sense if highly conserved genes enriched for essential functions have been integrated into more networks due to both their longer residence in the genome and greater regulatory pressure. By this line of reasoning, the risk genes for schizophrenia may be less interactive than MDD risk genes because the schizophrenia risk genes evolved more recently in humans—consistent with the lower conservation rate—resulting in weaker integration into ancient gene networks. Second, gene interaction networks may amplify the small individual effect sizes of risk variants via network ripple effects (51). This could occur through joint regulation of expression due to shared regulatory elements [e.g., promoters and enhancers (52)], similar local chromatin environments, and long-range linkage disequilibrium (53, 54), which could promote concomitant inheritance of risk burden. We predict that the greater the network ripple effects among risk genes, the fewer total risk variants will be required to meet threshold for manifesting a heritable disorder.

The phenotypes associated with the MDD risk genes may provide insights into disease mechanisms. We noticed that they fell broadly into three categories depending on when their effects on organism development were evident: early (Sterile, Embryonic), middle (Development, non-embryonic), or late (Locomotion, Neurotransmitter). If this reflects the situation in humans, it would suggest that genetic liability for MDD unfolds over the lifetime of the person. This would also allow for a richer interaction between genes and the environment, which will be very different going from *in utero* stages to early childhood upbringing and eventually to the stresses and challenges of adult life. Brain development and growth, including growth factors such as BDNF, were common themes for the functional activities of syntenic blocks of genes similar to other studies (55–57). Moreover, our data support earlier work concerning functional networks/pathways involved in MDD despite using very different approaches. Wong et al. (58) previously reported that MDD genes were focused on development and growth factor signaling, whereas Wray et al. (23) highlighted the role of the risk genes in brain development, synaptic function and gene regulation. Zhao et al. (59) identified protein-protein interaction networks that intersected with many of the genes and pathways discussed here including BDNF, other growth factors and Akt, a pivotal kinase that signals downstream of growth factors and insulin. Finally, variation in MDD risk gene counterparts in *C. elegans* produced significantly more alterations in life span than variation in genomic genes. This observation is consistent with the fact that MDD is associated with a significant decrease in life expectancy through both natural causes, such as cardiovascular disease, and suicide (33, 60, 61).

Several limitations to this study are worth discussing. The master list that we created is likely to include some false positives. This possibility plagues all GWAS analyses and other studies aimed at risk gene identification. It was a major motivation to obtain collateral information that would enhance confidence in the risk genes discovered so far. In addition, the risk genes were identified based on proximity to a genetic variant such as a single nucleotide polymorphism (SNP). Many enhancers and promoters are known to be far removed from the genes they regulate (62, 63) so choosing the genes closest to a SNP may miss relevant functional targets further away. Again, this is part of the reason for undertaking this effort; additional expression quantitative trait loci (eQTL) data might help to refine the search for the genes affected by a risk variant. The genetic interaction networks generated for this work with GeneMANIA relied on one extensive database compiled by Lin et al. (36). Genetic interactions obtained with alternative methods may have given a different picture. Despite this limitation, novel patterns emerged from this analysis that were distinguishable from interaction networks obtained with randomly-selected genes. Finally, our conclusions concerning the syntenic blocks of genes should be considered preliminary at this point pending independent support from other studies.

We are particularly intrigued by the content of three syntenic blocks of risk genes. Block 14 is an extended grouping and includes a dopamine receptor, tyrosine hydroxylase, insulin, IGF2 and proteins that regulate insulin secretion. Insulin resistance and alterations in IGF-1 and IGF-2 have been implicated in MDD (64–66) and may explain the three- to four-fold increase in depression in patients with diabetes (67, 68). Furthermore, insulin has recently been shown to regulate motivation to find food in *C. elegans* (69). Absence of insulin signaling in this species produces a diminished motivation phenotype upon food deprivation characterized by immobility similar to that observed in the forced swim test—an established model of depressive symptoms in rodents (70). Previously, we likened the immobility response to suicidal behavior (71) because animals will stay in place until they die rather than search for food, even though they are capable of movement. Interestingly, this same phenotype is produced in the CX5156 *sad-1(ky289)* strain (D.S.D, unpublished observation); *sad-1* is the *C. elegans* ortholog of BRSK2 located in extended block 14 (Table 1). As noted above, blocks 3 and 11 both contain CRB and DENND1 genes plus members of the LHX family of transcription factors. The fact that separate studies identified these same blocks of genes increases the likelihood of their relevance to MDD. Moreover, the *C. elegans* ortholog of LHX9 is *ttx-3*. The OH161 *ttx-3(ot22)* strain shows a similar “suicide” or immobility phenotype in our system that is corrected with antidepressant drugs (D.S.D., unpublished observation). It is worth noting that LHX6 has been identified near a suicide risk variant (72) and DCC, CACNA1C, NTRK2, RERE, and CKB from Supplementary Table 1 are possible suicide risk genes (73). When combined with the results showing conservation of MDD risk genes in *C. elegans* and zebrafish and the sharing of phenotypes associated with these genes, this work demonstrates the value of characterizing human disease risk genes in model organisms. Of course, additional studies will be necessary to confirm the significance of the molecular pathways proposed here and to translate these findings into potential treatments for MDD.

## Data Availability Statement

The original contributions generated for the study are included in the article/Supplementary Material, further inquiries can be directed to the corresponding author/s.

## Author Contributions

DD conceived of the project, supervised research efforts, performed statistical analyses and wrote the initial draft of the paper. SS, WT, and AS generated the gene lists, collected the data, helped to write, and revise the manuscript. All authors contributed to the article and approved the submitted version.

## Conflict of Interest

The authors declare that the research was conducted in the absence of any commercial or financial relationships that could be construed as a potential conflict of interest.
